# Sodium imaging of the heart at 7T: design, evaluation and application of a four-channel transmit/receive surface coil array

**DOI:** 10.1186/1532-429X-15-S1-W14

**Published:** 2013-01-30

**Authors:** A Graessl, A Ruehle, W Renz, L Winter, H Pfeiffer, J Ruff, J Rieger, T Niendorf

**Affiliations:** 1 Max-Delbrueck-Center for Molecular Medicine, Berlin, Germany; 2 Siemens Healthcare, Erlangen, Germany; 3 Physikalisch-Technische Bundesanstalt (PTB), Berlin, Germany; 4 Experimental and Clinical Research Center (ECRC), Charité Campus Buch, Humboldt-University, Berlin, Germany

## Background

Insight of physiological processes and cellular metabolism makes 23Na-MRI conceptually appealing as non-invasive imaging discipline. Several studies report the applicability of 23Na-MRI for the detection and assessment of acute and chronic heart disease due to increased sodium concentration after myocardial infarctions. Bi-exponential decay of the signal and a low SNR compared to 1H-MRI makes 23Na-MRI unattractive for clinical use. With a high SNR and fast imaging technologies ultrahigh field MRI brings 23Na-MRI back into focus, asking for dedicated radiofrequency (RF) technology.

## Methods

**Figure 1:**
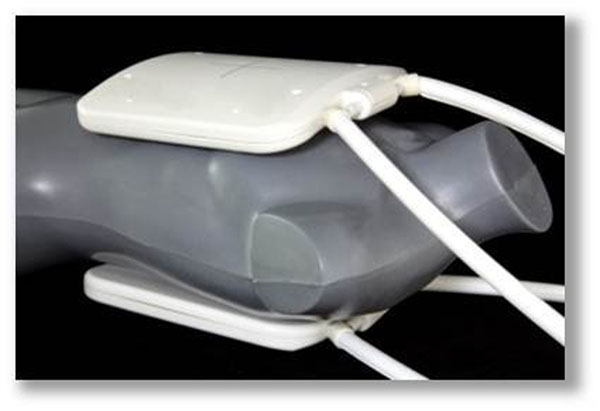
The coil housed in two modestly curved lightweight formers to conform to an average chest and back, shown on a mannequin.

The proposed four channel transceiver RF-coil tuned to 78.6 MHz consists of an anterior and a posterior section (Fig 1), each containing two 210 x 140 mm rectangular loop elements connected with a shared conductor. EMF and SAR simulations were performed using CST Studio Suite 2011 (CST AG, Darmstadt, Germany) together with the voxel model Duke from the Virtual family (ITIS Foundation, Zurich, Switzerland). In-vivo studies were performed in healthy volunteers using a 7 T whole body MRI system (Magnetom, Siemens, Erlangen, Germany). The volunteers were positioned prone to reduce artifacts due to respiratory motion. 23Na localizer imaging was performed with untriggered gradient echo (2D FLASH) imaging (FOV 320x380, TE=2,28 ms, TR=5,6 ms; TA=1,32 min; matrix size 5x5x40 mm, averages 256). Short axis images were acquired with a cardiac triggered gradient echo (2D FLASH) sequence (FOV 282x379, TE=2,24 ms, TR=200 ms; TA=11,23 min; matrix size 5,4x5,4x40 mm, averages 400) of the heart. For triggering an acoustic cardiac gating device (easyACT, MRI.Tools GmbH, Germany) was used.

**Figure 2:**
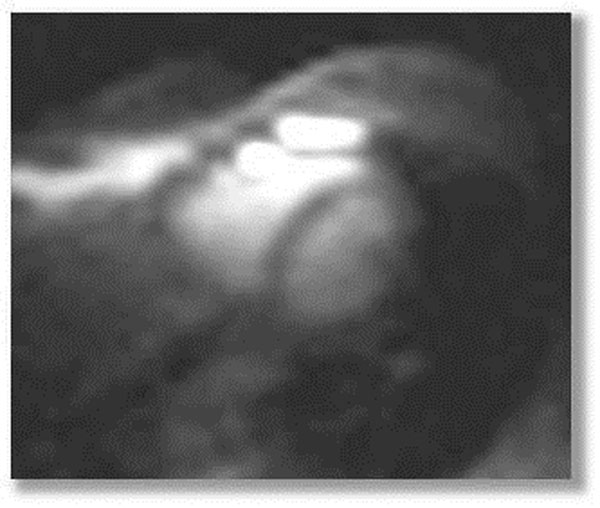
23Na short axis view of the heart acquired with an cardiac triggered 2D FLASH sequence.

## Results

The reflection coefficient of each element was measured to be better than -18 dB, transmission coefficients were found to be below -16 dB. The SAR values fell well within the limits provided by the IEC 60601-2-33 for input power of 10W in normal mode. In vivo studies yielded a rather uniform signal intensity across the heart leading to adequate image quality. An intense signal caused by the high 23Na-concentration of the ribs was observed. The cardiac triggered acquisitions showed a SNR of 38 in the blood-pool and 24 in the septum resulting in a blood/myocardium contrast of 3,3.

## Conclusions

Our results demonstrate that 23Na-MRI of the heart is feasible at 7.0 T. The proposed RF coil design yielded adequate image quality within clinically acceptable scan times for free breathing, cardiac triggered acquisitions. Using an even larger number of TX/RX channels would help to further boost SNR and spatial resolution together with scan time shortening.

